# Jarring

**Published:** 1890-08

**Authors:** F. H. Lutze

**Affiliations:** Cheshire, N. Y.


### JARRING.

Editors of The Homœopathic Physician:—In your item on Jarring, in the July number, page 312, you omitted to mention the following:

Cinchona-off.: Sensation as if head would burst, with sleeplessness, worse from motion *or any jar;* better in a room and when opening the eyes. See Hering's *Condensed Materia Medica*, second edition, page 308.

F. H. Lutze.

Cheshire, N. Y., July 10th, 1890.

[We are much indebted to Dr. Lutze for calling our attention to so important a symptom omitted from our item on Jarring.—Eds.]

